# Proximal junctional kyphosis in Lenke 5 AIS patients: the important factor of pelvic incidence

**DOI:** 10.1186/s12891-021-04052-8

**Published:** 2021-02-15

**Authors:** Quan Zhou, Bowen Hu, Xi Yang, Yueming Song, Limin Liu, Linnan Wang, Lei Wang, Chunguang Zhou, Zhongjie Zhou, Peng Xiu, Liang Wang

**Affiliations:** Department of Orthopedic Surgery and Orthopedic Research Institute, West China Hospital, Sichuan University, NO. 37 GuoXue Road, 610041 Chengdu, Sichuan China

**Keywords:** Proximal junctional kyphosis, Adolescent idiopathic scoliosis, Thoracolumbar/lumbar curve, Posterior surgery, Pelvic incidences, Sagittal alignment

## Abstract

**Background:**

The purpose of the study was to investigate whether pelvic incidence (PI) will affect the occurrence of PJK in Lenke 5 AIS patients after correction surgery and try to explore a better surgical scheme based on PI.

**Methods:**

Lenke 5C AIS patients that underwent correction surgery with a minimum of a 2-year follow-up were identified. Demographic and radiographic data were collected preoperatively, postoperatively, and at the final follow-up. The comparison between the PJK and the Non-PJK group was conducted and the subgroup analysis was performed based on the preoperative value of PI to investigate the potential mechanism of PJK. Clinical assessments were performed using the Scoliosis Research Society (SRS)-22 questionnaire.

**Results:**

The mean preoperative Cobb angle of the TL/L curve was 53.4°±8.6. At the final follow-up, the mean TL/L Cobb angle was drastically decreased to 7.3°±6.8 (*P* < 0.001). The incidence of PJK in Lenke 5 AIS was 18.6 %, 21.9 % (7/32) in the low PI group (PI < 45°) and 15.8 % (6/38) in the high PI group (PI ≥ 45°), and there was no statistical difference between the two groups (χ^2^ = 0.425, *P* = 0.514). For low PI patients, there is no significant difference where the UIV is located with regards to the TK apex between the PJK and Non-PJK subgroups (χ^2^ = 1.103, *P* = 0.401). For high PI patients, PJK was more likely to occur when UIV was cephalad to than caudal to the TK apex (31.25 % vs. 4.7 %, *P* = 0.038). There was no significant difference in the selection of LIV between the two groups.

**Conclusions:**

There is no difference in the incidence of PJK between the Lenke 5 AIS patients with low PI (< 45°) and high PI (≥45°), but the main risk factor of PJK should be different. For patients with low PI, overcorrection of LL should be strictly avoided during surgery. For patients with high PI, the selection of UIV should not be at or cephalad to the apex of thoracic kyphosis to retain more mobile thoracic segments.

## Background

Sagittal alignment is increasingly recognized as a critical parameter in the setting of spinal deformity [1]. For adolescent idiopathic scoliosis (AIS) patients, correction surgery is recommended to achieve a well-balanced spine on the coronal and sagittal alignment while improving appearance and quality of life. It is clear now that a sagittal alignment plays a major role in clinical outcomes and should not be neglected in AIS [2, 3]. Proximal junctional kyphosis (PJK), which is defined as the final proximal junctional sagittal Cobb angle (PJA) between the lower endplate of the uppermost instrumented vertebra (UIV) and the upper endplate of UIV + 2, which is ≥ 10°and at least 10°greater than the preoperative measurement [4], is one of the most common sagittal malalignment complications in AIS patients after surgery. The prevalence of the PJK after posterior surgery in AIS patients was reported as being between 7 % and 46 % in variation on different surgical methods or instrument types [5–7].

To date, a large number of studies have focused on PJK and divided the etiology into three categories: patient-specific, surgical, and radiographic risk factors. The patient-specific factors included gender and BMI, while the surgical factors included thoracoplasty and instrument types [8, 9]. Besides, some studies documented that the PJK was closely related to some sagittal alignment parameters, such as preoperative thoracic kyphosis (TK) > 40° [7, 10], PJA change > 5° [11], or substantial reduction of TK [10]. PJK should be considered as being the result of the comprehensive effect of multiple factors rather than a single factor. However, the mechanism of PJK is still not clear, and these risk factors are also controversial.

Previous studies have reported several preoperative or postoperative sagittal parameters, such as larger preoperative TK, larger postoperative LL, which are associated with PJK. Additionally, significant correlations between spinal sagittal parameters and the morphological pelvic incidence (PI) have been proved and pelvic parameters are the cornerstone in sagittal alignment regulation [12]. PI is described as a morphological parameter, not affected by the posture or the pelvis position and thought to remain constant until roughly 10 years of age [1, 13, 14]. The presence of abnormal PI is a risk factor for sagittal malalignment following scoliosis fusion surgery, which may lead to decreased quality of life and increased severity of symptoms [15, 16]. Christopher et al. [17] demonstrated that low PI was associated with increased PJK when using growth rod in early-onset scoliosis (EOS) patients. However, Emmanuelle et al. [5] found that patients with high PI were more at risk of PJK. Does the value of PI affect the occurrence of PJK after AIS correction surgery? To the best of our knowledge, the clinical outcome, natural course and compensatory mechanism of PJK in AIS patients are still yet to be fully understood. The purpose of the study is to investigate the potential mechanism of PJK in Lenke 5 AIS patients after correction surgery and to make better surgical plans based on PI.

## Materials and Methods

### Patients population

This retrospective study was conducted after the approval of the institutional review board (IRB). The data of Lenke 5 C AIS patients, operated between January 2010 and June 2017 for thoracolumbar/lumbar (TL/L) curves, were retrospectively analyzed. The inclusion criteria were as follows: (1) a diagnosis of Lenke 5 C AIS and Cobb angle of the TL/L curve of more than 40 °, (2) age: between 12 and 20 years, (3) patients treated with one-stage posterior all-pedicle screw instrumentation without adjuvant anterior release, (4) a minimum of a 2-year follow-up. Therefore, patients who had previous spine surgery, but without a minimum of a 2-year follow-up were excluded from this study. And Clinical examination and investigations were done to rule out any other cause of scoliosis.

### Radiographic Assessment

Standing full-length posteroanterior (PA), lateral radiographs by the multipurpose Digital R/F System (Sonialvision Safire 17; Shimadzu Corp., Kyoto, Japan) were performed routinely before surgery, immediately after the operation and at each follow-up time point. All radiological parameters were measured by 3 attending spinal surgeons who were not involved in the surgery, and the average value was adopted.

The Cobb angle of the main TL/L coronal curve was measured on the standing full-length radiographs. We presumed that the Cobb angle to be reliably measured needed to be within 5°. The preoperative grade of the Risser sign was also recorded and evaluated. On the lateral radiographs, the following sagittal parameters were measured: pelvic incidence (PI), sacral slope (SS), pelvic tilt (PT), lumbar lordosis (LL), thoracic kyphosis (TK, Cobb angle between T5-T12), global TK (GTK, Cobb angle between T1-T12), sagittal vertical axis (SVA), PI-LL (calculated by subtracting LL from PI), thoracolumbar junction kyphosis angle (TLK, Cobb angle between T10-L2), PJA.

PI, SS, and PT were measured using previously described standard methods [18]. The LL was the angle between the upper endplate of L1 and the superior endplate of S1. The TK was the angle from the superior endplate of T5 and the inferior endplate of T12, and GTK was the angle from the superior endplate of T1 and the inferior endplate of T12. SVA was the distance between the C7 plumb line and the posterosuperior corner of S1. A positive value indicated that the C7 plumb line was anterior to the posterior sacral corner, while a negative value indicated that the C7 plumb line was posterior to the posterior sacral corner. The PJA was measured as the angle between the inferior endplate of the UIV to the superior endplate of UIV + 2. For LL and TK, the positive means lordotic and negative means kyphotic. Lower PI, PT, and SS were found in Chinese children compared with published studies of adolescents in other countries [19–23]. Therefore, patients were divided into low PI (PI < 45°) and high PI (PI ≥ 45°) groups based on pelvic incidence.

All surgical procedures were performed by two attending spinal surgeons using the third-generation spinal instrumentation system. The upper instrumented vertebra (UIV) was chosen based on the side bending Cobb angle of the main thoracic spine. For those with main thoracic side bending Cobb angle < 15゜, the TL/L fusion was performed. For patients with main thoracic curve > 40° or side bending Cobb angle > 15°, the UIV could be extended within the compensatory main thoracic curve. Generally, UIV should be avoided as the apex of the thoracic kyphosis. Normally, the lowest instrumented vertebra (LIV) should end at least at lower end vertebra (LEV). Occasionally, LIV needs to be LEV + 1 to obtain a higher correction rate, especially for patients with TL/L Cobb angle more than 60°. The rod bending during the operation was based on the surgeons’ personal experience. Clinical assessments were performed using the Scoliosis Research Society (SRS)-22 questionnaire.

### Statistical analysis

All data were analyzed by using SPSS 24.0 software (SPSS Inc., Chicago, IL). Results are presented as mean ± standard deviation for continuous variables and as number (percentage) for categorical variables. The normal distribution of the data was demonstrated with the Shapiro–Wilk test. Student t-test (for normally distributed data) and Mann-Whitney-Wilcoxon test (for nonparametric data) were used to determine the statistical differences in continuous data, whereas chi-square test was performed for categorical data analyses. The threshold for statistical significance was a p-value < 0.05.

## Results

In total, 70 Lenke 5 AIS patients (16 males and 54 females) were included for analysis from the database. The average age of the whole sample was 15.3 ± 2.1 years at the time of surgery. The mean preoperative Cobb angle of the TL/L curve was 53.4°±8.6. At the final follow-up, the mean TL/L Cobb angle was drastically decreased to 7.3°±6.8 (*P* < 0.001). The mean correction rate of the TL/L curve was 86.5 %±11.5 %. The mean follow-up was 36.7 ± 15.5 months. There was no significant difference in age, Risser sign, gender distribution, follow-up time.

PJK occurred in 13 out of 70 patients (18.6 %) until the final follow-up, while the remainder of the 57 patients demonstrated no PJK. Whether PJK occurred or not, patients were divided into PJK (*n* = 13) and Non-PJK groups (*n* = 57). Subgroup analysis was performed between the TL/L fusion and extended fusion to the thoracic curve, and PJK was comparable between the two groups (*P* = 0.322). The preoperative TL/L Cobb angle and the correction of the Cobb angle were not statistically different between both groups. The PJK group had larger TK, GTK, LL, and PI-LL mismatch than the Non-PJK group. The detailed statistical results of radiological parameters between the two groups are shown in Table 1. The most common UIV were T7 (*n* = 13), followed by T6 (*n* = 12) and T9 (*n* = 12), T8 (*n* = 11), T10 (*n* = 8), T5 (*n* = 7), T4 (*n* = 4), T11 (*n* = 2) and T12 (*n* = 1). The most common LIV were L4 (*n* = 32), followed by L3 and L5 (*n* = 19 each). No significant differences were noted in the selection of UIV (*P* = 0.946) and LIV (*P* = 0.680) between the high PI and low PI groups, respectively.

**Table 1:** Comparison of radiological parameters between the PJK group and the Non-PJK group

	Total (*n* = 70)	Non-PJK Group (*n* = 57)	PJK Group (*n* = 13)	P#
TL/L Cobb angle				
Preoperative	53.4 ± 8.6	53.9 ± 8.8	51.5 ± 7.9	0.374
Postoperative	6.5 ± 5.9	6.3 ± 6.1	7.5 ± 5.4	0.507
Final	7.3 ± 6.8	7.1 ± 6.9	8.4 ± 6.3	0.515
PI	45.5 ± 10.2	46.1 ± 10.1	43.1 ± 10.8	0.352
SS				
Preoperative	38.0 ± 7.7	37.8 ± 7.6	38.7 ± 8.4	0.724
Postoperative	36.2 ± 6.9	36.3 ± 7.0	35.7 ± 6.4	0.761
Final	36.9 ± 7.5	37.0 ± 7.7	37.3 ± 6.5	0.827
PT				
Preoperative	7.5 ± 6.9	8.2 ± 6.6	4.4 ± 7.9	0.077
Postoperative	9.3 ± 7.7	9.8 ± 7.0	7.1 ± 10.3	0.247
Final	8.7 ± 7.4	9.3 ± 7.0	5.9 ± 8.4	0.132
LL				
Preoperative	52.0 ± 10.7	50.7 ± 10.5	57.7 ± 10.2	**0.031**
Postoperative	50.9 ± 9.0	50.1 ± 8.6	56.4 ± 8.9	**0.015**
Final	52.6 ± 9.5	51.1 ± 9.1	58.8 ± 8.9	**0.006**
GTK (T1-12)				
Preoperative	28.3 ± 12.9	26.2 ± 12.0	37.6 ± 12.9	**0.003**
Postoperative	27.1 ± 11.3	25.3 ± 11.4	37.2 ± 6.3	**0.004**
Final	32.1 ± 12.9	29.6 ± 12.1	42.8 ± 10.8	**< 0.001**
TK (T5-12)				
Preoperative	21.1 ± 10.7	18.9 ± 8.7	30.7 ± 13.4	**0.001**
Postoperative	20.4 ± 8.9	17.7 ± 8.5	29.3 ± 5.7	**0.001**
Final	24.3 ± 11.9	21.6 ± 9.4	36.0 ± 14.8	**< 0.001**
SVA (mm)				
Preoperative	-6.9 ± 27.7	-6.5 ± 26.4	-8.5 ± 34.5	0.825
Postoperative	-9.1 ± 23.2	-10.3 ± 22.6	-3.8 ± 26.1	0.371
Final	-10.7 ± 24.7	-10.9 ± 24.7	-9.5 ± 25.4	0.844
PJA				
Preoperative	9.3 ± 4.0	9.3 ± 4.3	8.8 ± 2.4	0.636
Postoperative	11.1 ± 5.7	9.9 ± 5.1	16.2 ± 5.2	**< 0.001**
Final	14.9 ± 8.0	12.3 ± 5.3	26.3 ± 7.6	**< 0.001**
PI-LL				
Preoperative	-6.5 ± 11.9	-4.6 ± 10.9	-14.6 ± 13.1	**0.005**
Postoperative	-5.4 ± 10.3	-3.6 ± 8.8	-13.6 ± 12.4	**0.001**
Final	-7.0 ± 10.0	-5.0 ± 8.8	-15.7 ± 10.3	**< 0.001**

*PJK* proximal junctional kyphosis; *TL/L* thoracolumbar/lumbar; *PI* pelvic incidence; *LL* lumbar lordosis; *GTK* global thoracic kyphosis; *TK* thoracic kyphosis; *SVA* sagittal vertical axis; *PJA* proximal junctional angle

“#” comparison between the PJK group and the Non-PJK group

Subgroup analysis was performed according to the value of PI to investigate the potential mechanism of PJK. Our results showed that the incidence of PJK was 21.9 % (7/32) in the low PI group (PI < 45°) and 15.8 % (6/38) in the high PI group (PI ≥ 45°), and there was no statistical difference between the two groups (χ^2^ = 0.425, *P* = 0.514). The high PI group had larger PT, SS, and LL than the low PI group, but there was no significant difference in TK, GTK, SVA, and PJA (Table 2). A comparison of radiological parameters between the Non-PJK and PJK subgroups with low PI was shown in Table 3. Significant differences were found in LL, TK, GTK, PI-LL between the PJK and Non-PJK subgroups. In these low PI patients, the UIV levels were as follows: T8 and T9 (n = 6 each), T10, T6, T7 (n = 5 each), T5 (n = 3), and T4 (n = 2). There is no significant difference where the UIV is located with regards to the apex of thoracic kyphosis between the PJK and Non-PJK subgroups (χ^2^ = 1.103, *P* = 0.401) (Table 3). There was no statistical difference in the selection of LIV (χ^2^ = 0.969, *P* = 0.738).

**Table 2:** Comparison of radiological parameters between the low PI group and the high PI group

Variables	Low PI (<45°) *n*=32	High PI (≥45°) *n*=38	p
PJK	*n*=7	*n*=6	0.514
TL/L Cobb angle			
Preoperative	52.53±8.96	54.14±8.38	0.443
Postoperative	6.2±5.4	6.8±6.4	0.690
Final	7.1±6.3	7.6±7.3	0.746
PI	37.1±6.0	52.6±7.2	**<0.001**
SS			
Preoperative	33.0±5.5	42.2±6.7	**<0.001**
Postoperative	32.0±4.7	39.8±6.4	**<0.001**
Final	32.1±6.1	40.9±6.0	**<0.001**
PT			
Preoperative	4.1±6.5	10.4±6.0	**<0.001**
Postoperative	5.1±8.1	12.8±6.3	**<0.001**
Final	5.2±8.1	11.7±5.2	**<0.001**
LL			
Preoperative	49.2±11.4	54.4±9.7	**0.043**
Postoperative	47.5±7.9	53.9±8.9	**0.002**
Final	47.6±7.6	56.7±9.0	**<0.001**
GTK (T1-12)			
Preoperative	28.4±14.1	28.3±12.1	0.997
Postoperative	28.5±10.2	29.7±12.3	0.666
Final	30.5±11.9	33.5±13.6	0.334
TK (T5-12)			
Preoperative	22.6±12.9	19.9±8.5	0.285
Postoperative	22.7±9.0	20.42±8.9	0.299
Final	24.6±11.1	24.1±12.8	0.868
SVA			
Preoperative	-10.5±30.7	-3.9±25.1	0.330
Postoperative	-8.6±26.5	-9.6±20.5	0.886
Final	-13.1±21.2	-8.7±27.5	0.466
PJA			
Preoperative	9.3±3.7	9.3±4.4	0.949
Postoperative	11.5±5.5	10.7±5.9	0.539
Final	14.8±8.9	15.1±7.2	0.894
PI-LL			
Preoperative	-12.1±12.3	-1.7±9.3	**<0.001**
Postoperative	-10.4±10.8	-1.3±7.8	**<0.001**
Final	-10.4±10.2	-4.2±9.0	**0.009**

*PJK* proximal junctional kyphosis; *TL/L* thoracolumbar/lumbar; *PI* pelvic incidence; *LL* lumbar lordosis; *GTK* global thoracic kyphosis; *TK* thoracic kyphosis; *SVA* sagittal vertical axis; *PJA* proximal junctional angle

**Table 3:** Comparison of radiological parameters between PJK and non-PJK patients with low PI

Low PI (PI < 45°) *n*=32	Non-PJK Group *n*=25	PJK Group *n*=7	p
PI	37.8±6.2	34.3±4.4	0.169
SS			
Preoperative	33.1±6.2	32.7±1.6	0.688
Postoperative	31.5±4.5	33.5±5.5	0.336
Final	31.4±6.3	34.4±5.4	0.266
PT			
Preoperative	4.7±6.8	1.6±5.2	0.267
Postoperative	6.4±6.8	0.7±6.8	0.059
Final	6.6±8.3	0.1±5.1	0.059
LL			
Preoperative	47.3±11.4	55.5±8.9	0.095
Postoperative	45.7±6.9	53.6±8.5	**0.017**
Final	45.5±6.2	54.8±7.7	**0.003**
Δ LL (post-pre)	-1.6±11.4	-1.8±9.0	0.958
Δ LL (final-pre)	-1.8±11.0	-0.6±7.7	0.804
GTK (T1-12)			
Preoperative	24.5±11.8	42.2±13.4	**0.002**
Postoperative	25.9±9.6	37.8±6.0	**0.004**
Final	27.4±10.9	41.5±8.7	**0.004**
Δ T1-12 (post-pre)	1.3±10.9	-4.3±15.6	0.273
Δ T1-12 (final-pre)	2.9±9.9	-0.7±13.3	0.438
TK (T5-12)			
Preoperative	18.8±9.1	36.2±15.8	**0.001**
Postoperative	17.3±8.2	31.0±6.6	**0.004**
Final	21.3±8.7	36.2±11.4	**0.001**
Δ T5-12 (post-pre)	-1.4±8.1	-5.1±13.1	0.105
ΔT5-12 (final-pre)	2.4±8.0	0.01±11.0	0.514
SVA (mm)			
Preoperative	-9.2±30.5	-15.2±33.	0.654
Postoperative	-11.4±25.5	1.5±29.5	0.262
Final	-15.4±20.4	-4.8±23.5	0.251
PJA			
Preoperative	9.3±4.0	9.2±1.8	0.941
Postoperative	9.9±4.5	17.1±5.2	**0.001**
Final	11.3±5.2	27.2±8.1	**<0.001**
Δ PJA (final-pre)	1.9±4.2	18.0±7.7	**<0.001**
PI-LL			
Preoperative	-9.5±11.5	-21.1±10.9	**0.024**
Postoperative	-7.8±9.5	-19.4±10.6	**0.009**
Final	-7.5±8.7	-20.3±9.3	**0.002**
Δ PI-LL (post-pre)	1.7±11.0	1.7±9.0	0.987
Δ PI-LL (final-pre)	1.9±10.8	0.8±7.8	0.804
UIV			0.401
UIV above apex of TK	9	4	
UIV below apex of TK	16	3	

*PJK* proximal junctional kyphosis; *PI* pelvic incidence; *SS* sacral slope; *PT* pelvic tilt; *LL* lumbar lordosis; *GTK* global thoracic kyphosis; *TK* thoracic kyphosis; *SVA* sagittal vertical axis; *PJA* proximal junctional angle; *UIV* upper instrumented vertebra

Table 4 summarized preoperative, postoperative, and final follow-up radiological parameters of the high PI patients. The PJK subgroup had larger final GTK (*P* = 0.030), TK *P* = 0.012), and LL (*P* = 0.003) than the Non-PJK subgroup at the final follow-up. The value of PI-LL was within the ideal range before and after surgery (|PI-LL| <10°). Although the change of TK was larger in the PJK subgroup after surgery, there was no statistical difference between the two subgroups. The UIV levels were as follows: T7 (*n* = 8), T6 (*n* = 7), T9 (*n* = 6), T8 (*n* = 5), T5 (*n* = 4), T10 (*n* = 3), T4 and T11 (*n *= 2 each), T12 (*n* = 1). Our results showed that PJK was more likely to occur when UIV was cephalad to than caudal to the TK apex (31.25 % vs. 4.7 %, *P* = 0.038) (Table 4). However, there was no significant difference in the selection of LIV (χ^2^ = 2.153, *P* = 0.390).

**Table 4:** Comparison of radiological parameters between PJK and non-PJK patients with high PI

High PI (≥45°) *n*=38	Non-PJK Group *n*=32	PJK Group *n*=6	p
PI	52.5±7.6	53.4±4.9	0.779
SS			
Preoperative	41.6±6.4	45.7±7.7	0.169
Postoperative	40.1±6.4	38.2±7.0	0.524
Final	41.0±6.0	40.7±6.4	0.912
PT			
Preoperative	10.9±5.1	7.7±9.6	0.461
Postoperative	12.5±5.9	14.5±8.7	0.482
Final	11.5±5.1	12.7±6.2	0.604
LL			
Preoperative	53.2±9.0	60.3±11.7	0.098
Postoperative	52.8±8.9	59.5±8.9	0.086
Final	55.3±8.6	63.8±8.3	**0.003**
Δ LL (post-pre)	-0.4±7.9	-0.7±15.2	0.927
Δ LL (final-pre)	2.1±8.1	3.4±14.4	0.756
GTK (T1-12)			
Preoperative	27.6±12.2	32.2±11.1	0.394
Postoperative	28.3±12.7	36.4±7.0	0.143
Final	35.4±12.7	44.4±13.5	**0.030**
Δ T1-12 (post-pre)	0.7±7.9	4.20±7.31	0.337
ΔT1-12 (final-pre)	7.8±8.3	12.1±15.7	0.061
TK (T5-12)			
Preoperative	19.0±8.5	24.3±6.6	0.163
Postoperative	17.8±8.9	22.2±3.9	**0.036**
Final	28.8±10.1	35.8±19.3	**0.012**
Δ T5-12 (post-pre)	-1.2±7.6	-2.1±7.7	0.409
ΔT5-12 (final-pre)	9.8±9.7	11.5±19.7	0.336
SVA (mm)			
Preoperative	-4.5±22.9	-0.7±37.1	0.732
Postoperative	-9.5±20.5	-10.1±22.5	0.946
Final	-7.6±27.6	-14.9±28.6	0.555
PJA			
Preoperative	9.4±4.5	8.3±3.0	0.568
Postoperative	9.8±5.6	15.2±5.5	**0.036**
Final	13.1±5.3	25.3±7.5	**<0.001**
Δ PJA (final-pre)	3.6±3.8	17.0±8.0	**<0.001**
PI-LL			
Preoperative	-0.7±8.6	-6.9±11.7	0.135
Postoperative	-0.2±6.6	-6.8±11.3	0.220
Final	-2.9±8.5	-10.4±9.2	0.058
UIV			**0.038**
UIV above apex of TK	11	5	
UIV below apex of TK	21	1	

*PJK* proximal junctional kyphosis; *PI* pelvic incidence; *SS* sacral slope; *PT* pelvic tilt; *LL* lumbar lordosis; *GTK* global thoracic kyphosis; *TK* thoracic kyphosis; *SVA* sagittal vertical axis; *PJA* proximal junctional angle

The clinical outcomes using the SRS-22 questionnaires were shown in Table 5. No significant differences were found between the PJK and Non-PJK groups in the SRS-22 domain scores for function/activity, pain, self-image/appearance, mental health, or satisfaction with management. The self-image/appearance and mental health domain score demonstrated statistically significant difference at the final follow-up compared with preoperative data (*P* < 0.01). There was no intraoperative neuromonitoring alert, no neurologic complication in this cohort. None of the patients underwent revision surgery because of PJK.

**Table 5:** Preoperative and final follow-up for SRS-22 Domain scores

	All cohorts	Non-PJK (*n*=57)	PJK (*n*=13)	p
Function/activity				
Preoperative	4.2±0.38	4.1±0.26	4.2±0.32	0.54
Final follow up	4.2±0.56	4.2±0.44	4.1±0.62	0.37
Pain				
Preoperative	4.1±0.46	4.0±0.35	4.2±0.39	0.26
Final follow up	4.2±0.22	4.1±0.41	4.2±0.33	0.20
Self-image/appearance				
Preoperative	3.2±0.44	3.1±0.50	3.3±0.49	0.41
Final follow up	3.9±0.47*	3.9±0.52*	3.8±0.36*	0.66
Mental health				
Preoperative	3.4±0.35	3.5±0.49	3.3±0.34	0.82
Final follow up	3.9±0.47*	4.0±0.63*	3.9±0.52*	0.43
Satisfaction with management	4.2±0.22	4.1±0.21	4.2±0.23	0.14
SRS-22 Total (final follow-up)	4.1±0.39	4.1±0.40	4.0±0.37	0.77

*Significantly different from the preoperative value, *p*<0.01.

## Discussion

Our results showed that the incidence of PJK was 18.6 %, and stood in the lower range of the literature for similar patients [5, 10, 24]. Previous studies have found that greater correction of LL resulted in higher incidence of PJK and they attributed surgical overcorrection of sagittal deformities as a risk factor [25–27]. Kim et al. [25] identified that excessive lordosis and larger sagittal balance correction led to PJK, requiring revision surgery. Lafage et al. [26] recently refocused the attention of PJK prevention onto spinal alignment, by providing age-adjusted sagittal alignment parameters for adults. They also reported that with increased overcorrection beyond age-adjusted sagittal alignment there is a corresponding increase in PJK severity. PJK is more likely to occur after sagittal alignment (like LL) overcorrection, which is accepted by most researchers. Our clinical experience suggests that LL overcorrection is more likely to occur in patients with a low PI. Up to now, there are no reports on the influence of PI value on PJK in Lenke 5 AIS patients.

Pelvic incidence remains relatively constant during childhood and it determines the pelvic orientation and the size of the lordosis, which is closely correlated with it [18]. In short, the greater the PI, the greater has to be SS, PT, or both. The PI value varies widely among individuals and it is related to the capacity to compensate for sagittal alignment. Our results showed that there was no significant difference in the incidence of PJK between the high and low PI group patients, but the mechanism of PJK was different among high PI and low PI patients. Chinese children and adolescents had lower PI, PT, and SS compared with the Caucasian population [19–21]. In our previous study [22], the mean value of PI of Chinese adolescents was about 45°, so we chose the value of 45° as the critical value of high PI and low PI.

**Fig. 1:**
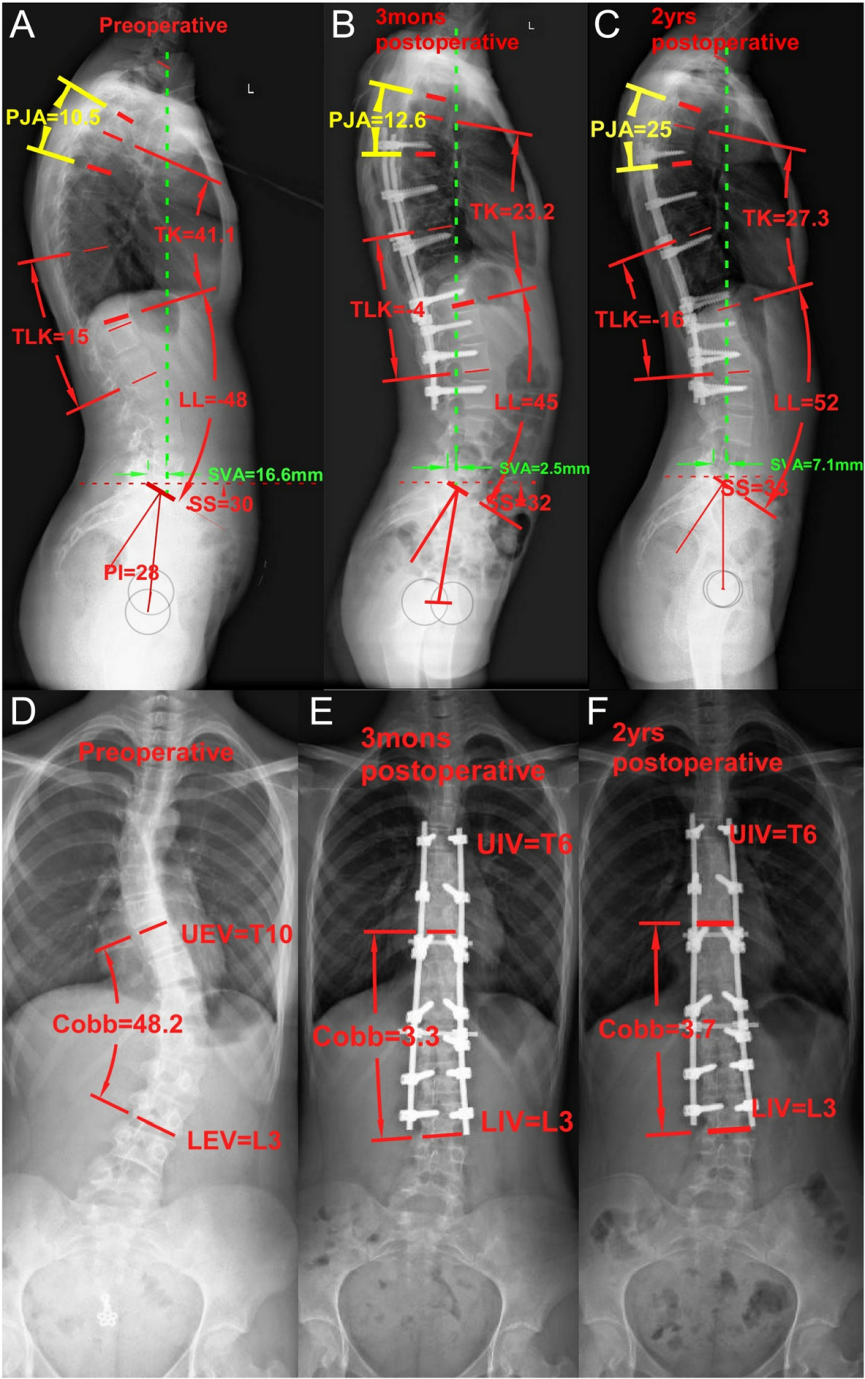
The pre-and postoperative radiographs of a 15-year-old female patient with low PI (PI = 28°) who developed PJK after surgery. She underwent T6-L3 instrumentation. The thoracolumbar junction is corrected from kyphosis to lordosis. Postoperative TK was significantly reduced, while the PJA was significantly increased and PJK occurred at 2 years’ follow-up. Other sagittal parameters did not change significantly (**a** to **c**). In the coronal plane, the major thoracolumbar curve was corrected from 48.2° to 3.3°, which remained stable (3.7°) at a two-year follow-up. The UIV was T6 and the LIV was L3 (**d** to **f**). The apex of thoracic kyphosis was T7 (**a**). PI, pelvic incidence; SS, sacral slope; LL, lumbar lordosis; TK, thoracic kyphosis; GTK, global thoracic kyphosis; PJA, proximal junctional angle; TLK, thoracolumbar junctional kyphosis angle; SVA, sagittal vertical axis

For the patients with low PI, The PJK patients have a larger postoperative LL and worse PI-LL than the Non-PJK patients. However, for patients with high PI, PJK patients have a similar PI-LL to Non-PJK patients. Roussouly et al. advocated that the ability to retrovert the pelvis is limited by a patient’s PI, and patients with a low PI have a small capacity to compensate for their sagittal imbalance through pelvic retroversion [28]. In the circumstance of low PI, if the surgical procedure introduces more lumbar lordosis than the patient’s PI can accommodate, then the thoracic spine would begin to join into compensation mechanism with additional thoracic kyphosis (PJA↑), which may result in PJK (Fig. 1). Patients with low PI have poor pelvic compensation to abnormal sagittal alignment. In such low PI cases, the overcorrection of lumbar lordosis after surgery would easily exceed pelvic compensation. Therefore, postoperative LL increase was more likely to be compensated by increased proximal kyphosis above the fusion levels. For the reasons mentioned above, it is reasonable to assume that low PI patients require strictly matched postoperative LL.

**Fig. 2:**
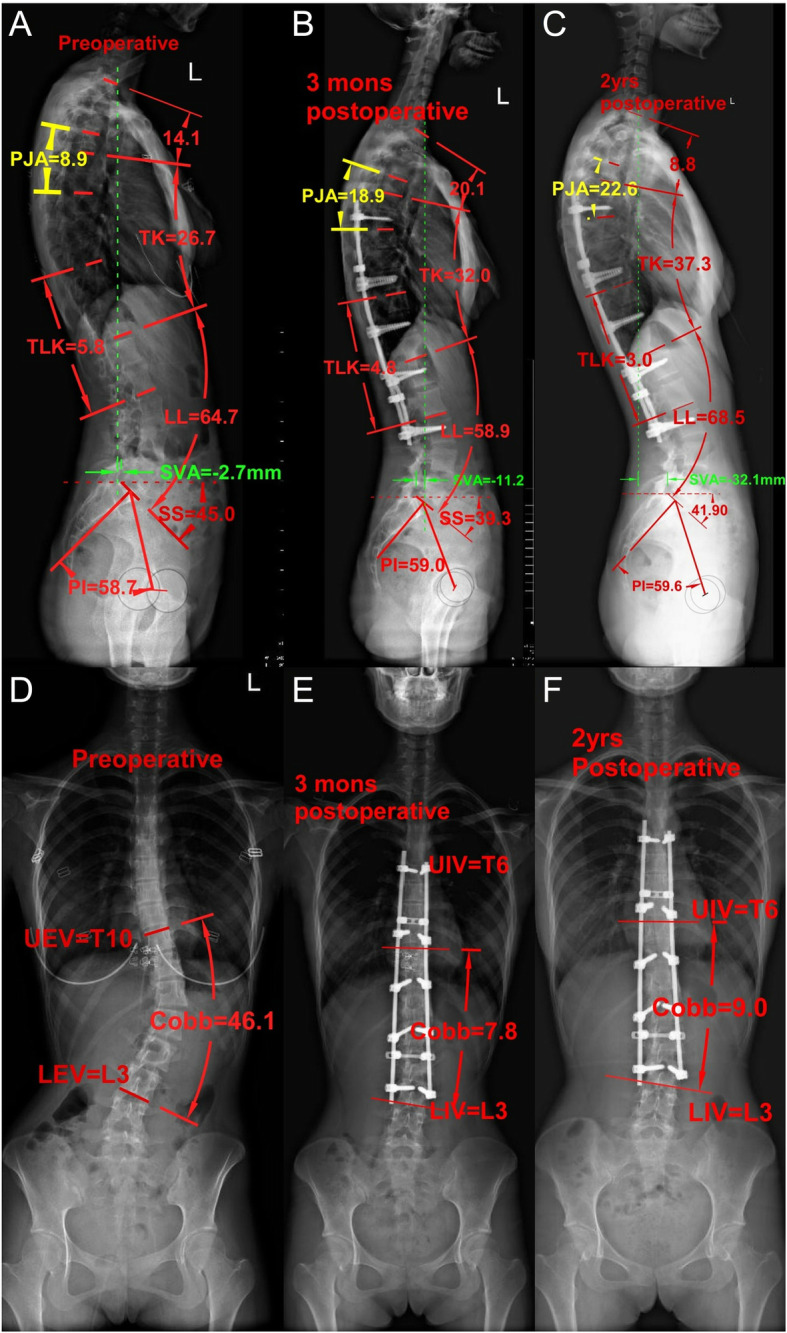
The pre-and postoperative radiographs of a 17-year-old female patient with high PI (PI = 58.7°) who developed PJK after surgery. She underwent a T6-L3 fusion. The PJA increased to 22.6° at 2 years’ follow-up (**a** to **c**). The thoracolumbar/lumbar curve was corrected from 46.1°to 7.8° (**d** to **e**). The UIV was T6 and the LIV was L3. The apex of thoracic kyphosis was T7. PI, pelvic incidence; SS, sacral slope; LL, lumbar lordosis; TK, thoracic kyphosis; GTK, global thoracic kyphosis; PJA, proximal junctional angle; TLK, thoracolumbar junctional kyphosis angle; SVA, sagittal vertical axis

In contrast, patients with high PI had larger PT, SS, LL. These results are consistent with previously published articles [12, 29]. In this study, we found no statistically significant differences in features on preoperative radiological findings between the PJK and Non-PJK subgroups with high PI. The PI-LL were all within 10° in the high PI group before and after surgery, which means PI and LL match well. Even though LL did not meet the ideal curvature at the time of surgery, the remaining lumbar segments below LIV could compensate. It is well known that AIS patients generally have a flat back. In particular, posterior column osteotomy may further reduce TK. To keep well-matched between PI and LL, TK and LL, patients will have a compensatory increase in TK after surgery. Patients increased their proximal kyphosis above UIV to compensate for the postoperative decrease in instrumented TK and to balance head over the pelvis, which introduces PJK (Fig. 2). The higher the UIV selection, the fewer remaining thoracic segments that can compensate for TK reduction. The risk of PJK may increase in this situation. Our current findings revealed that the UIV in the PJK group was more likely to extend across the apex of TK, which was consistent with the above compensatory mechanism. In practice, there remains no clear consensus regarding the optimal LIV and UIV in individual patients. The selection of fusion levels continues to vary widely based on patient, regional, and surgeon variables. Therefore, more prospective studies or multi-center studies are needed to verify our results.

For Lenke 5 C AIS patients, we suggest that the surgical procedure should be designed according to the PI to minimize the incidence of PJK: the conventional posterior column osteotomy will potentially increase LL. For patients with low PI, overcorrection of LL should be strictly avoided during surgery. We recommend that we should not bend too much lordosis in the lumbar level and begin bending the lordosis below L3 or L4. For patients with high PI, the selection of UIV should try not to cross the apex of thoracic kyphosis to retain more mobile thoracic segments. Lonner et al. [30] also found that UIV at or cephalad to the UEV was the only significant risk factor for PJK in patients with Lenke type 5 curves in the multivariate analysis model. Their results showed that patients with UIV at or cephalad to UEV were 9.1 times more likely to develop PJK at 2 years after surgery. If UIV selection was inevitable too high, we suggest to increase the density of the screws in the thoracic level and try to bend more TK to correct the flat-back deformity in such case.

Several limitations still exist. Firstly, it was a retrospective study and had a relatively small sample size. Statistically, a high UIV seems to expose high PI patients to a significant risk of PJK but the significance of this finding remains unclear and requires further investigations. Further analysis, such as a randomized trial with a larger number of patients, will be necessary to verify our findings. Secondly, even though there was no significant association between the PJK and SRS-22 scores in this young population. However, these were only short-term follow-up results, and the influence of PJK on long-term quality of life for AIS patients still needs to be further observed.

## Conclusions

The incidence of PJK in Lenke 5 AIS was 18.6 %. Our results found no difference in the incidence of PJK according to PI, but the mechanisms of PJK may be different for different PI values. Patients with low PI have a small capacity to compensate for their sagittal imbalance, if the surgical procedure introduces more lumbar lordosis (LL overcorrection) than the patient’s PI can accommodate, then the proximal unfused segments may compensate more LL through PJK. Patients with high PI have a large compensatory capacity through the pelvis, but if the UIV was too high, which limits the overall compensation of the spine and pelvis and may lead to PJK. The design of surgical plans for Lenke 5 AIS patients should take individual PI into account to decrease the incidence of PJK. For patients with low PI, overcorrection of LL should be strictly avoided during surgery. For patients with high PI, the selection of UIV should not be too high to retain more mobile thoracic segments.

## Data Availability

The datasets used during the current study are available from the corresponding author on reasonable request.

## Abbreviations

AIS: Adolescent idiopathic scoliosis
PJK: Proximal junctional kyphosis
PJA: Proximal junctional sagittal Cobb angle
UIV: The upper instrumented vertebra
LIV: Lowest instrumented vertebra
TK: Thoracic kyphosis
LL: Lumbar lordosis
PI: Pelvic incidence
EOS: Early-onset scoliosis
IRB: The institutional review board
TL/L: Thoracolumbar/lumbar
PA: Posteroanterior
SS: Sacral slope
PT: Pelvic tilt
GTK: Global TK (T1-T12 sagittal Cobb angle)
SVA: Sagittal vertical axis
TLK: Thoracolumbar junction kyphosis angle (Cobb angle between T10-L2)
ANOVA: Analysis of variance
HRQOL: Health-Related Quality of Life
